# LFformer: An improved Transformer model for wind power prediction

**DOI:** 10.1371/journal.pone.0309676

**Published:** 2024-10-25

**Authors:** Dongjin Ma, Yingcai Gao, Qin Dai

**Affiliations:** 1 State Key Laboratory of Low-carbon Smart Coal-fired Power Generation and Ultra-clean Emission, China Energy Science and Technology Research Institute Co., Ltd., Nanjing, Jiangsu, China; 2 Shenyang Institute of Engineering, Shenyang, Liaoning, China; Australian Catholic University, AUSTRALIA

## Abstract

Wind power forecasting has complex nonlinear features and behavioral patterns across time scales, which is a severe test for traditional forecasting techniques. To address the multi-scale problem in wind power forecasting, this paper innovatively proposes an ultra-short-term forecasting model LFformer based on Legendre-Fourier, which firstly focuses on the important information in the input sequences by using the encoder-decoder architecture, and then scales the range of the original data with the Devlin normalization method, and then utilizes the Legendre polynomials to The data sequence is projected into a bounded dimensional space, the historical data is compressed using feature representation, then feature selection is performed using the low-rank approximation method of Fourier Transform, the prediction is inputted into the multilayer perceptron through the multi-scale mixing mechanism, and finally the results are outputted after back-normalization. The experimental results show that compared with the existing prediction methods, the model realizes the improvement of prediction accuracy and stability, especially in the ultra-short-term prediction scenario, with obvious advantages. The research results are not only valuable for improving the overall operational efficiency of the wind power system, but also help to enhance the stable operation of the power grid, which provides strong technical support and guarantee for wind power enterprises to improve the competitiveness of bidding for Internet access in the power market competition.

## Introduction

With the growing global demand for clean energy, wind power, as an important part of renewable energy, is becoming increasingly important. The latest Global Renewable Energy Outlook (GREO) report [1] released by the International Energy Agency (IEA) points out that wind power is expected to account for nearly one-third of the global electricity supply by 2050, becoming a key force in promoting the transformation of the energy structure [2, 3].

However, the stochastic and intermittent nature of wind brings great challenges to the power prediction of wind power generation, which affects the stable operation of the power system and the effective scheduling of the power market. Therefore, improving the accuracy and reliability of wind power generation power prediction is of great significance for ensuring grid security, promoting renewable energy consumption, and reducing energy costs [4, 5].

In addition, with the advancement of smart grid construction, the requirements for real-time and multi-scale wind power prediction are getting higher and higher, and the exploration of new prediction models and methods to improve the comprehensive performance of wind power prediction has become a hot spot and a difficult point in current research [6].

In recent years, research for wind power prediction has made significant progress, and deep learning techniques based on Transformer [7] and its variants have demonstrated powerful performance in sequence prediction tasks. The Transformer model captures the long-term dependencies in the data through the self-attention mechanism, which effectively improves the accuracy and efficiency of prediction [8, 9]. However, the computational complexity of the traditional Transformer model increases significantly with the increase of sequence length, and there is the problem of high consumption of computational resources. In addition, in the wind power prediction task, meteorological factors such as wind speed have multi-scale characteristics, and the Transformer model has certain limitations in capturing these multi-scale features.

Aiming at the shortcomings of Transformer in dealing with long series data, the Machine Learning Group of Tsinghua University School of Software (THUML-LAB) proposes Autoformer [10], a long term series prediction model based on a deep decomposition architecture and an autocorrelation mechanism, which uses the Auto-Correlation Mechanism [11] instead of the Auto-attention Mechanism, and embedded the decomposition of data in the model structure through the Deep Decomposition Architecture, which improved the model’s ability to handle long series data. However, Autoformer still relies on the autocorrelation mechanism in capturing multi-scale features, and may not be able to fully utilize the changing patterns of meteorological factors, such as wind speed, over different time scales.

Similarly, in order to deal with long-series time series data, Zhou HY et al. proposed an Informer [12] model with lower time complexity and space complexity, which effectively reduces computational resource consumption and improves the model’s prediction performance through the introduction of sparsity processing techniques and sequence decomposition and reorganization techniques. However, in wind power prediction, the multiscale characteristics of wind speed and other meteorological factors have an important impact on the prediction accuracy, and the model needs to be further optimized to better capture the multiscale features.

In addition, there are many improved models based on the Transformer model [13, 14], such as the NHits [15] model proposed by Cristian Challu et al. which mainly focuses on the time series prediction task and captures the different scale features of the time series data through the hierarchical interpolation structure of neural networks; the Performer [16] model proposed by Krzysztof et al. model by Krzysztof et al. approximates the traditional dot product attention by introducing a kernel method, which reduces the computational complexity; and the Reformer [17] model by Nikita Kitaev et al. optimizes the Transformer model by techniques such as locally-sensitive hashing (LSH) attention mechanism and reversible residual network, which reduces the computational complexity and memory footprint.

In view of the limitations of the multi-scale feature capturing ability of the above models leading to shortcomings in wind power prediction, this paper proposes a Legendre-Fourier-based multi-scale wind power prediction model, LFformer. by combining the Legendre polynomials [18] and the Fourier transform [19], the model is able to deal with both local details and global trends in the wind power time series, realize the effective fusion of multi-scale information, and improve the prediction accuracy and generalization ability. Specifically, the work done in this paper is as follows:

1. propose a multi-scale wind power prediction model based on Legendre polynomials and Fourier transform, LFformer, which can effectively extract and fuse multi-scale time series features to improve the prediction accuracy and generalization ability.
2. introduce a projection module based on Legendre polynomials, which can be integrated into various types of time series prediction models, effectively solving the problem of retaining and utilizing historical information in long-term prediction, and providing support for complex time series analysis in wind power prediction tasks.
3. propose a frequency enhancement layer to reduce the dimensionality by combining Fourier analysis and low-rank matrix approximation to minimize the influence of noise signals in time series and alleviate the problem of over-extraction.
4. introduce a data normalization method based on statistical properties to scale the numerical range of the original data to a specific interval, in order to eliminate the influence of the wind power generation data with varying magnitudes and large differences in numerical ranges on the model training.

## LFformer model

**Fig 1:**
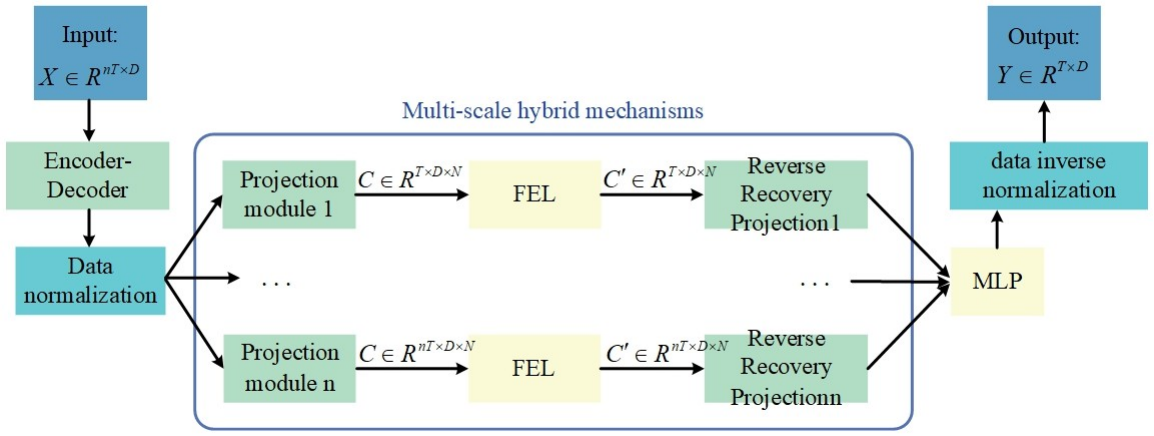
The overall structure of LFformer model.

In order to reduce the information redundancy and improve the independence and separability of the data for subsequent operations such as feature extraction, dimensionality reduction and frequency domain analysis, this paper uses Legendre polynomials to form the projection module. Meanwhile, in order to capture the frequency features in the time series more effectively, this paper uses a convolution layer based on Fourier transform to perform convolution operations in the frequency domain to capture the frequency features of the input data, so that the model can analyze the periodicity and trend in the time series data more effectively. In addition, in order to enhance the robustness of the model, this paper uses the data normalization method proposed by Devlin et al [20] to normalize the input data and denormalize it before output. The overall structure of the model is shown in Fig 1. The input data are first normalized, then enter the projection module to project into the Legendre polynomial space C, and then processed by the frequency enhancement layer to generate the, and finally, the output sequence is obtained by inverse recovery projection and denormalization. Finally, the prediction is performed by a multilayer perceptron (MLP) and the predictions are back-normalized and output. In this case, the multiscale mixing mechanism [21] utilizes input sequences of different time spans {T, 2T, …, nT} to predict the span T and merges each prediction with a linear layer.

### Encoder-decoder

**Fig 2:**
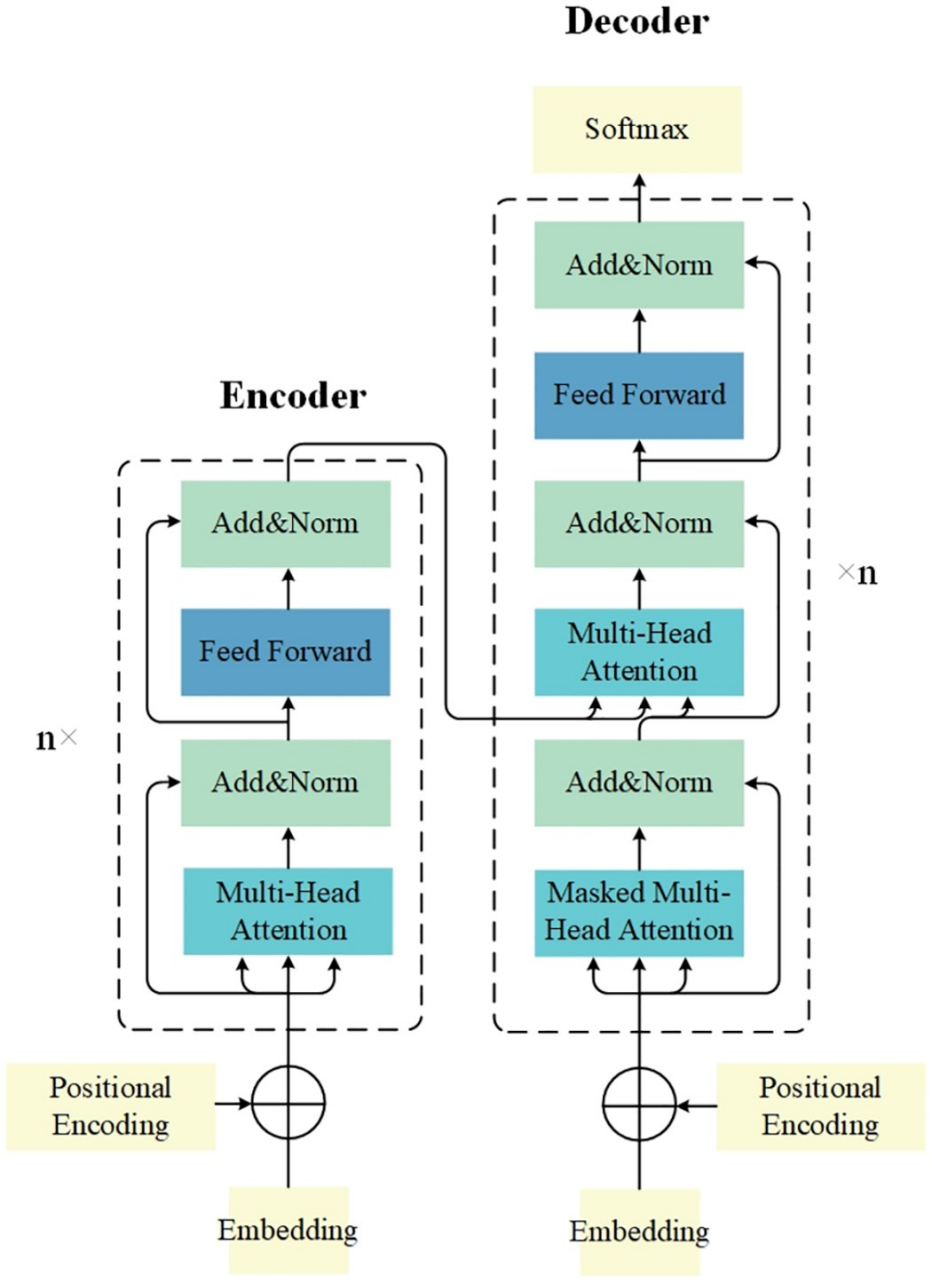
Encoder-decoder structure.

In order to enhance the ability of the model to pay attention to the input sequences when generating the output, and to improve the performance and efficiency of the model in processing the sequence data, this paper introduces the encoder-decoder architecture of Transformer, whose structure is shown in Fig 2.

Among them, the encoder consists of a stack of 6 identical layers, each with 2 sub-layers. The first sublayer is a multi-head self-attention mechanism and the second sublayer is a positionally fully connected feedforward network. Residual connections are used around each of the two sublayers, followed by layer normalization. The output of each sublayer is, where Sublayer(x) is a function realized by the sublayer itself. To facilitate residual joins, all sublayers in the model as well as the embedding layer produce outputs of dimension.

The decoder also consists of a stack of six identical layers. In addition to the two sub-layers in each encoder layer, the decoder inserts a third sub-layer that performs multi-head attention on the output of the encoder stack. Similar to the encoder, residual concatenation is used around each sublayer followed by layer normalization.

### Projection module

In order to decompose the input data into different frequency components for subsequent analysis and processing, this paper uses Legendre polynomials, with different orders of Legendre polynomials corresponding to different frequency components, to form a projection module that projects the data series onto a bounded dimensional space, thus providing a compressed or featured representation of the evolving historical data.

In this paper, Legendre polynomials of highest order N-1 are used to build the function *g*^(*t*)^(*x*):

$$\begin{array}{c} g^{\left(t\right)}\left(x\right)=\sum_{n=1}^{N}c_{n}\left(t\right)P_{n}\left(\frac{2(x-t)}{\theta }+1\right) \end{array}$$ (1)

where *P*_*n*_() is a Legendre polynomial of order n and the coefficients *c*_*n*_(*t*) are given by the following dynamic equation:

$$\begin{array}{c} \frac{d}{dt}c\left(t\right)=-\frac{1}{\theta }Ac\left(t\right)+\frac{1}{\theta }Bf\left(t\right) \end{array}$$ (2)

where A and B are defined as described in the literature [22] as ideal state space matrices.

If f (x) satisfies the Lipschitz [23] condition, then $\|f_{\left[t-\theta ,t\right]}\left(x\right)-g^{\left(t\right)}\left(x\right){\|}_{\mu^{\left(t\right)}}\leq o\left(\theta L/\sqrt{N}\right)$, Furthermore, if there are bounded derivatives of order k, then $\|f_{\left[t-\theta ,t\right]}\left(x\right)-g^{\left(t\right)}\left(x\right){\|}_{\mu^{\left(t\right)}}\leq o\left(\theta^{k}N^{-k+1/2}\right)$.

It can be obtained that the larger the base of Legendre polynomial, the higher the accuracy of the approximation. However, the increase of the base of Legendre polynomials will lead to too much noise signal in the history, and longer data history may lead to more history noise accumulation, therefore, in this paper, a fixed window size is used for function approximation and feature extraction.

Let A be a unit matrix, *ε*_*t*_ is a *σ*^2^-dimensional Gaussian random noise, then $x_{t}=A^{\theta }x_{t-\theta }+\sum_{i=1}^{\theta -1}A^{i}b+o\left(\sigma \sqrt{\theta }\right)$, For accuracy, an autoregressive random noise is set up in this paper. Let the time series{*x*_*t*_} ∈ *R*^*d*^, we have *x*_*t*+1_ = *Ax*_*t*_ + *b* + *ε*_*t*_, (*t* = 1, 2, …), where *A* ∈ *R*^*d*×*d*^, *b* ∈ *R*^*d*^, *ε*_*t*_ ∈ *R*^*d*^ is the random noise sampled from *N*(0, *σ*^2^*I*). Given *x*_*t*_, noise will accumulate in *x*_*t*−*θ*_ at a rate $\sqrt{\theta }$, where *θ* is the window size.

**Fig 3:**
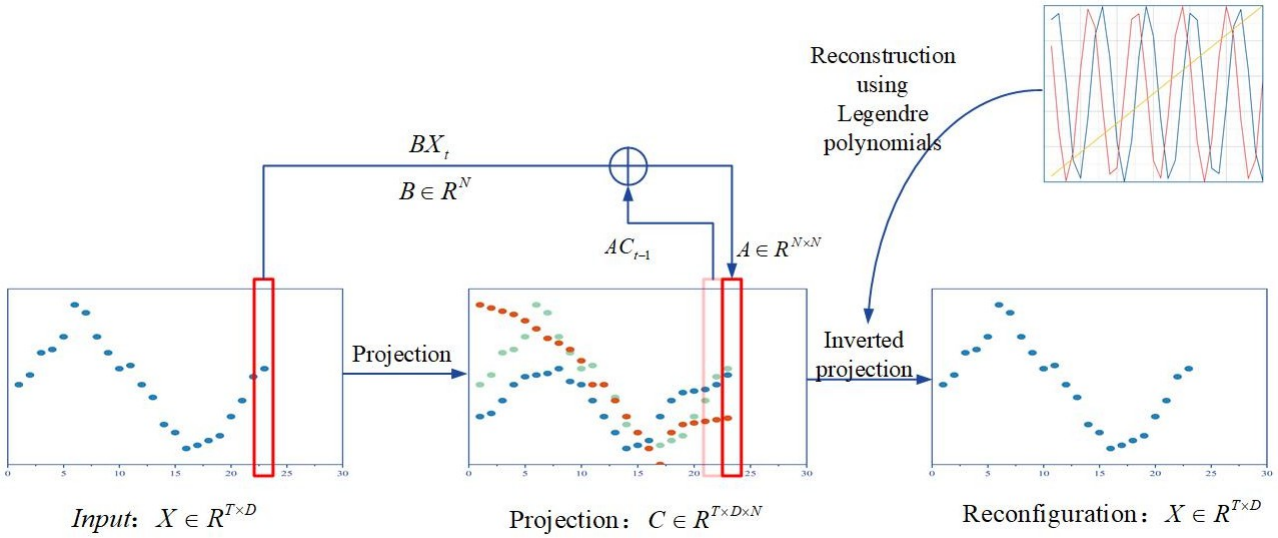
The structure of the projection module.

The structure of the projection module is shown in Fig 3. The principle is a state space model:

$$\begin{array}{c} C_{t}=AC_{t-1}+Bx_{t} \end{array}$$ (3)

Where *x*_*t*_ ∈ *R* is the input signal, *C*_*t*_ ∈ *R*^*N*^ is the storage cell, and N is the number of Legendre polynomials.

The projection module contains 2 preset matrices A and B, defined as follows:

$$A_{nk}=\left(2n+1\right)\left\{\begin{array}{ll} {\left(-1\right)}^{n-k} & \text{if}\;k\leq n\;\\ 1 & \text{if}\;k\geq n\; \end{array},\right.B_{n}=\left(2n+1\right){\left(-1\right)}^{n}$$ (4)

The projection module contains 2 phases, i.e., projection phase and reverse projection phase. The projection stage projects the original signal to the storage unit C and the reverse projection stage reconstructs the signal from the storage unit.

### Frequency enhancement layer

If the above features are directly input into deep learning modules such as MLP [24] and RNN [25] without screening, the performance of the model will not be improved due to the accumulation of historical noise. Therefore, this paper introduces a frequency enhancement layer and utilizes Fourier transform for feature selection.According to Eq. (1), the approximation function *g*^(*t*)^(*x*) can be stabilized by smoothing the coefficients *c*_*n*_(*t*). Since the smoothing of n can be simply achieved by multiplying the learnable scalar by each channel, only *c*_*n*_(*t*) in t needs to be smoothed by the Fourier transform.

Assume that the Fourier coefficient of *c*_*n*_(*t*) is *a*_*n*_(*t*).Based on the spectral bias, assuming the existence of *s*, *a*_min_ > 0, we have *t* > *s*, |*a*_*n*_(*t*)| ≤ *a*_min_ for all n. When sampling, the first k dimensions are kept and the remaining dimensions are sampled randomly.

Let *A* ∈ *R*^*d*×*n*^ be the matrix of Fourier coefficients of the input matrix *X* ∈ *R*^*d*×*n*^ and the consistency measure *μ*(*A*) = Ω(*k*/*n*) of the matrix A.Suppose that there exists a s and an *a*_min_ such that the element in the last d-s column of A is smaller than *a*_min_. If the first column is retained, randomly select *o*(*k*^2^/*ε*^2^ − *s*) columns from the remainder:

$$\begin{array}{c} \|A-P(A){\|}_{F}\leq o\left[\left(1+\varepsilon \right)a_{\text{min}}\cdot \sqrt{(n-s)d}\right] \end{array}$$ (5)

where P(A) denotes the matrix that projects A into the selected column space. When *a*_min_ is sufficiently small, the selected space can be considered almost identical to the original space.

**Fig 4:**
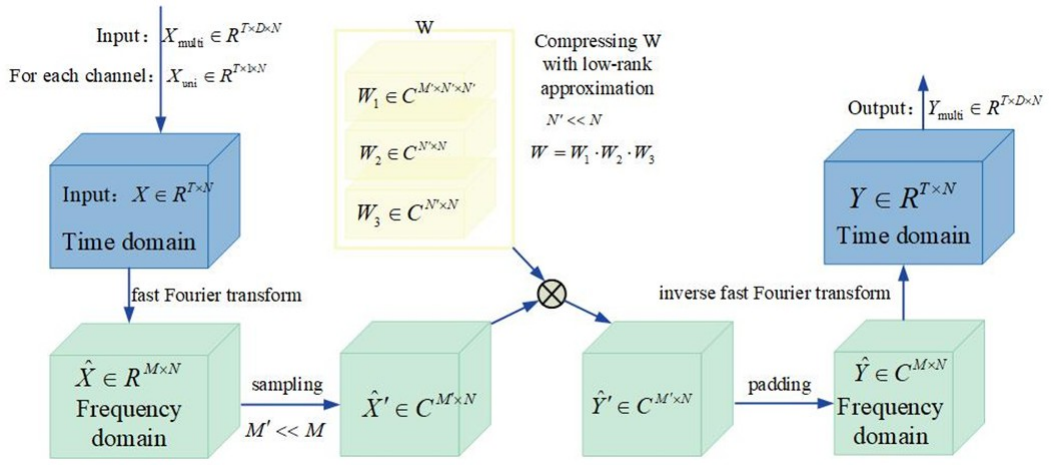
The structure of the frequency enhancement layer(FEL).

The structure of the frequency enhancement layer is shown in Fig 4. The entirety of what the model learns from the data forms a learnable weight matrix *W* ∈ *R*^*M*′×*N*×*N*^. To compress the size of the weight matrix, it is decomposed into three matrices:

$$\begin{array}{c} \begin{array}{c} W_{1}\in C^{M^{\prime }\times N^{\prime }\times N^{\prime }},\\ W_{2}\in C^{N^{\prime }\times N},\\ W_{3}\in C^{N^{\prime }\times N} \end{array} \end{array}$$ (6)

Perform a low-rank approximation(*N*′ < < *N*).

### Multilayer perceptron layers

**Fig 5:**
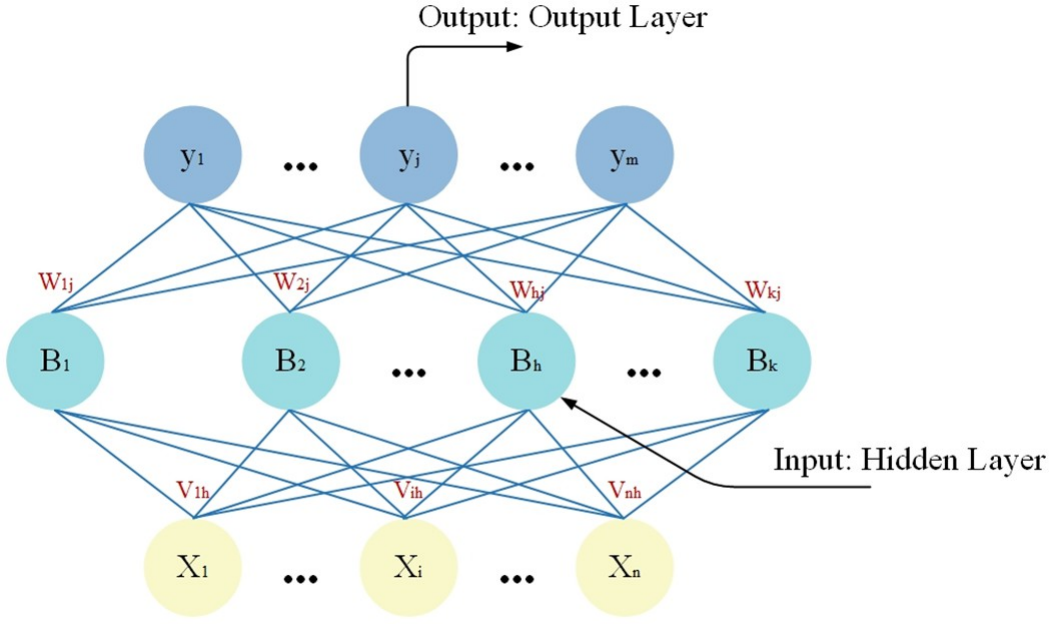
MLP forward propagation process.

The prediction process of multilayer perceptual machine (MLP) mainly consists of two stages, forward propagation and back propagation, but usually only forward propagation is involved in prediction, and the process is shown in Fig 5.

Given the data, where x is an n-dimensional vector and y is an m-dimensional vector, the MLP can be set up as a network structure with n input neurons, k hidden layer neurons (the number of k is variable) and m output layer neurons grouped into layers. Where the weight between the ith neuron of the output layer and the hth neuron of the hidden layer is assumed to be, and the weight between the hth neuron of the hidden layer and the jth neuron of the output layer is, then the input received by the hth neuron of the hidden layer is:

$$\begin{array}{c} \begin{array}{c} \alpha_{h}=\sum_{i=1}^{n}f(V_{ih}*X_{i}) \end{array} \end{array}$$ (7)

The input received by the jth neuron of the output layer is:

$$\begin{array}{c} \begin{array}{c} \beta_{j}=\sum_{h=1}^{m}f(W_{hj}*B_{i}) \end{array} \end{array}$$ (8)

Where is the output of the hth hidden layer neuron. Signaling is achieved by connecting the output of the previous layer to the input of the next layer.

### Data normalization

In order to make the input data have similar scales and accelerate the convergence speed, the input data are normalized in this paper. According to the literature [26], the mean and standard deviation of each instance $x_{k}^{\left(i\right)}\in R^{T}$ are calculated by the formula:

$$\begin{array}{c} \begin{array}{c} {\text{E}}_{\text{t}}\left[x_{kt}^{\left(i\right)}\right]=\frac{1}{T}\sum_{j=1}^{T}x_{kj}^{\left(i\right)},\text{Var}\left[x_{kt}^{\left(i\right)}\right]=\frac{1}{T}\sum_{j=1}^{T}{\left(x_{kj}^{\left(i\right)}-{\text{E}}_{\text{t}}\left[x_{kt}^{\left(i\right)}\right]\right)}^{2} \end{array} \end{array}$$ (9)

Using these statistics, the input data *x*^(*i*)^ is normalized to:

$$\begin{array}{c} \begin{array}{c} {\hat{x}}_{kt}^{\left(i\right)}=\gamma_{k}\left(\frac{x_{kt}^{\left(i\right)}-{\text{E}}_{\text{t}}\left[x_{kt}^{\left(i\right)}\right]}{\sqrt{\text{Var}[x_{kt}^{\left(i\right)}]+\varepsilon }}\right)+\beta_{k} \end{array} \end{array}$$ (10)

Where *γ*, *β* ∈ *R*^*K*^ is the learnable parameter vector. The normalized input data is fed to the model for prediction by the above operation. Finally, the model output is denormalized using the inverse of the normalization described above.

## Analysis of experimental results

### Data set

In this paper, power generation data from January 2, 2021 to June 23, 2022 from the wind power station of China Longyuan Power Group was used. The dataset contains data on predicted wind speed (WS), wind direction (WD), temperature (TEMP), humidity (HU), barometric pressure (PRES), system-generated predicted power (PREP), actual wind speed (A.WS.1), metering caliber1measured power (A.P,0), and actual power (YD15), with a sampling interval of 15 minutes.

### Data preprocessing

The training, validation and test sets were divided according to the ratio of 7:1:2. In order to select the features with strong correlation with the target variables for model training, and then improve the accuracy and efficiency of the model, this paper uses Pearson’s correlation coefficient [27] to calculate the strength and direction of the linear relationship between each influencing factor and power generation, and the formula of Pearson’s correlation coefficient r is as follows:

$$\begin{array}{c} \begin{array}{c} r=\frac{\text{cov}(X,Y)}{\sigma_{X}\times \sigma_{Y}} \end{array} \end{array}$$ (11)

where cov(*X*, *Y*) is the covariance of variables X and Y, and *σ*_*X*_ and *σ*_*Y*_ are the means of variables X and Y, respectively.

**Fig 6:**
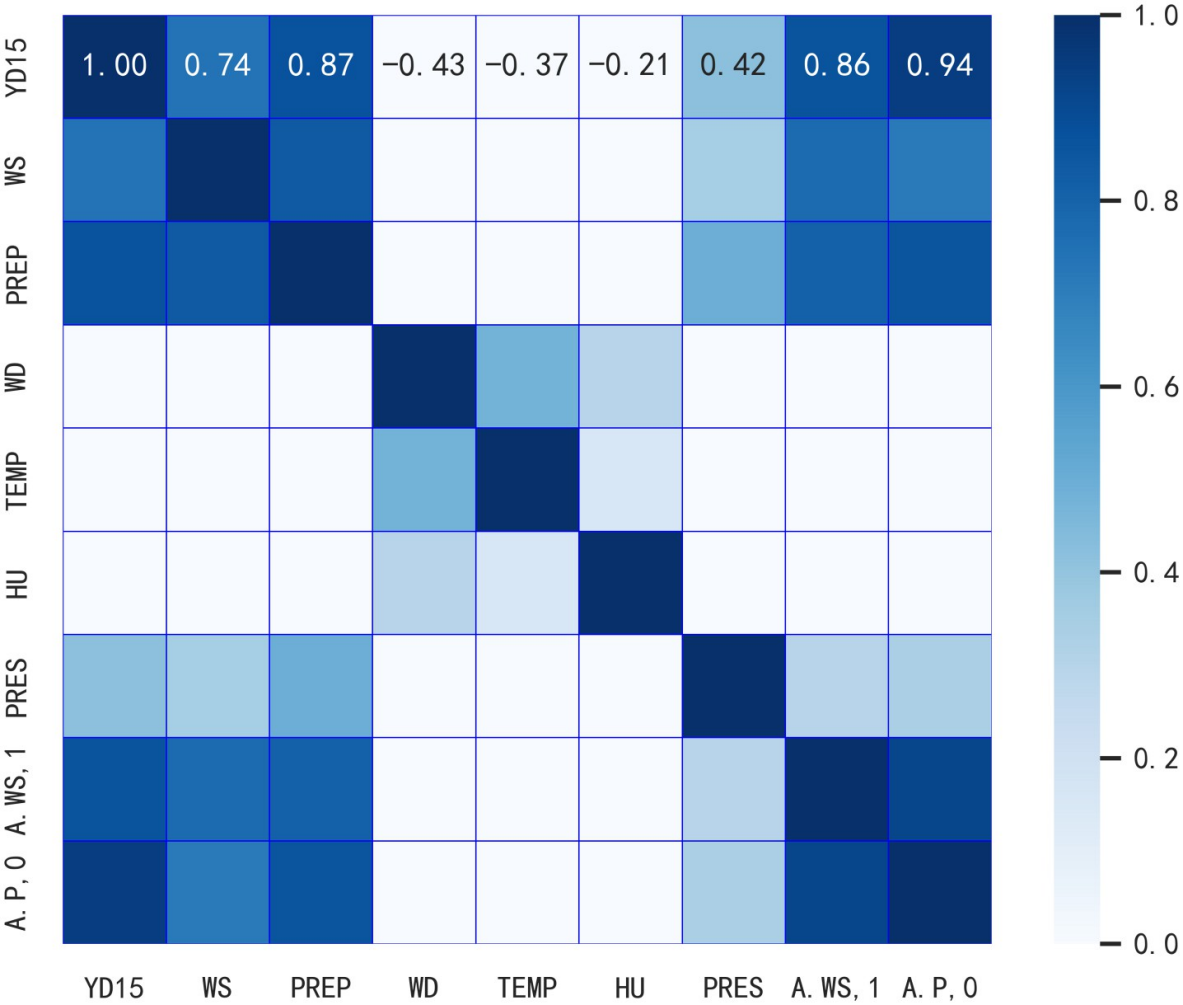
The correlation heat map.

After Pearson correlation analysis, the correlation heat map was obtained as shown in Fig 6:

The analysis reveals that wind direction (WD), temperature (TEMP) and humidity (HU) are negatively correlated with the actual power (YD15), so the wind direction, temperature and humidity features are deleted, and the forecast wind speed (WS), the projected power of the system (PREP), the barometric pressure (PRES), the actual wind speed (A.WS,1), and the actual generating power (YD15) are retained as input features to the model.

### Experimental environment and model evaluation indicators

The experimental platform used in this paper is based on NVIDIA A30 GPUs with 24 GB of video memory, Python version 3.10, using Pytorch 1.13.1 deep learning framework, and GPU CUDA version 11.4.

In evaluating the prediction performance of the model, a recognized evaluation index system in the field of new energy power generation prediction is adopted, specifically, four indexes, namely, the mean absolute error (*P*_*MAPE*_), the root mean square error (RMSE), the mean square error (MAE), and the mean absolute error (MSE), which are used to measure and quantify the prediction accuracy and stability of the model.

### Horizontal comparison experiment

Horizontal comparison experiments were conducted in order to assess the effect of a variable or set of variables by comparing results under different experimental conditions, thereby improving the accuracy and reproducibility of experimental results. First, model ‘F+MLP’ uses Fourier convolution module to extract features and then MLP neural network is used for power generation prediction. Second, model ‘L+F+MLP’ adds Legendre module to the head of model ‘F+MLP’. Finally, model ‘LFformer’ adds the Encoder-Decoder module at the head of model ‘L+F+MLP’. The performance comparison of the different models is shown in Tables 1 to 3 below.

**Table 1:** Horizontal comparison of ultra-short-term prediction.

model	P_MAPE_/%	RMSE/kW	MSE/kW	MAE/kW
Transformer	19.90	13743.47	1.89 × 10^8^	10612.43
F+MLP	25.97	21908.63	4.80 × 10^8^	18755.25
F+L+MLP	21.80	19988.55	4.00 × 10^8^	14930.34
LFformer	**15.97**	**11977.48**	**1.43** × **10**^**8**^	**10169.12**

Bolded data is optimal.

**Table 2:** Horizontal comparison of short-term(0–24 hours) prediction.

model	P_MAPE_/%	RMSE/kW	MSE/kW	MAE/kW
Transformer	480.34	29114.53	8.48 × 10^8^	20320.55
F+MLP	406.14	23558.05	5.55 × 10^8^	19986.56
F+L+MLP	273.82	**16509.72**	**2.73** × **10**^**8**^	**13476.91**
LFformer	**81.71**	21511.19	4.63 × 10^8^	17517.71

Bolded data is optimal.

**Table 3:** Horizontal comparison of short-term(0–72 hours) prediction.

model	P_MAPE_/%	RMSE/kW	MSE/kW	MAE/kW
Transformer	906.66	51259.11	2.63 × 10^9^	44257.91
F+MLP	588.82	29153.63	8.50 × 10^8^	24942.31
F+L+MLP	**430.35**	**22225.50**	**4.94** × **10**^**8**^	**18749.32**
LFformer	471.27	28092.05	7.89 × 10^8^	22024.00

Bolded data is optimal.

The data analysis reveals that incorporating the Fourier transform feature extraction module led to a slight decline in the model’s ultra-short-term forecasting performance, while there was an improvement in short-term predictions for both 0–24 hour and 0–72 hour horizons.

The inclusion of the Fourier transform feature extraction module leads to a degradation in the performance of ultrashort-term prediction due to the nature of the Fourier transform, which is more effective in capturing periodic or frequency-based patterns over longer time scales. In ultrashort-term forecasting, where the temporal dynamics are unstable and nonperiodic, the frequency characteristics introduced by the Fourier transform do not align well with rapidly changing data, leading to a decrease in accuracy.

However, for short-term forecasts of 0–24 hours and 0–72 hours, the ability of the Fourier transform to extract relevant frequency components becomes more favorable. Periodic trends and cyclical behavior are more pronounced in these time frames, allowing the model to utilize these features to make more accurate predictions. As a result, short-term prediction performance is improved as the model is better able to capture and utilize the underlying periodic structure in the data.

The addition of the Legendre module resulted in a further enhancement of predictive accuracy compared to the model utilizing only the Fourier transform feature extraction.

The improvement in prediction performance with the addition of the Legendre module compared to Fourier transform feature extraction alone can be attributed to the complementary nature of the two methods. The Fourier transform, which decomposes the time series into frequency components, is good at capturing global periodic patterns, but it can be difficult to accurately model local transient variations, which are crucial for accurate prediction, especially in non-stationary time series. The Legendre module, on the other hand, is adept at representing complex nonlinear patterns in data through the use of orthogonal polynomials. This allows it to capture finer local details in the time series that the Fourier transform may miss. By combining the global frequency characteristics of the Fourier transform with the local nonlinear modeling capabilities of the Legendre module, the model provides a more comprehensive view of the data. The synergy between the two modules improves the overall forecasting performance of the model, as it can now more effectively account for global cyclical trends and local variations.

Upon integrating the encoder-decoder module, the model achieved optimal performance in ultra-short-term predictions. Although the 0–24 hour and 0–72 hour forecasting performance experienced a slight reduction compared to the results with the Legendre module, it still outperformed the traditional Transformer model.

The introduction of the encoder-decoder module resulted in optimal performance for ultra-short-term predictions, which can be attributed to the module’s ability to efficiently capture and process temporal dependencies in sequential data. The encoder-decoder architecture is particularly well-suited for modeling complex temporal relationships by encoding input sequences into a latent representation that preserves relevant temporal information, and then decoding this representation to produce accurate forecasts. This capability is especially beneficial in ultra-short-term forecasting, where the rapid, high-frequency variations in the data require precise modeling of immediate past information.

However, the slight decline in predictive accuracy for the 0–24 hour and 0–72 hour short-term horizons, compared to the model without the encoder-decoder module, may be due to the module’s focus on optimizing ultra-short-term dynamics at the expense of capturing longer-term trends. While the encoder-decoder module excels at fine-tuning predictions in the immediate future, it may not fully leverage the periodic and frequency-based features that are more relevant for longer-term forecasts, as captured by the Fourier and Legendre modules.

Despite this trade-off, the model incorporating the encoder-decoder module still outperforms the traditional Transformer model. This can be attributed to the encoder-decoder’s more sophisticated handling of temporal dependencies, allowing it to provide more accurate predictions overall, even if the gains are more pronounced in the ultra-short-term range.

**Fig 7:**
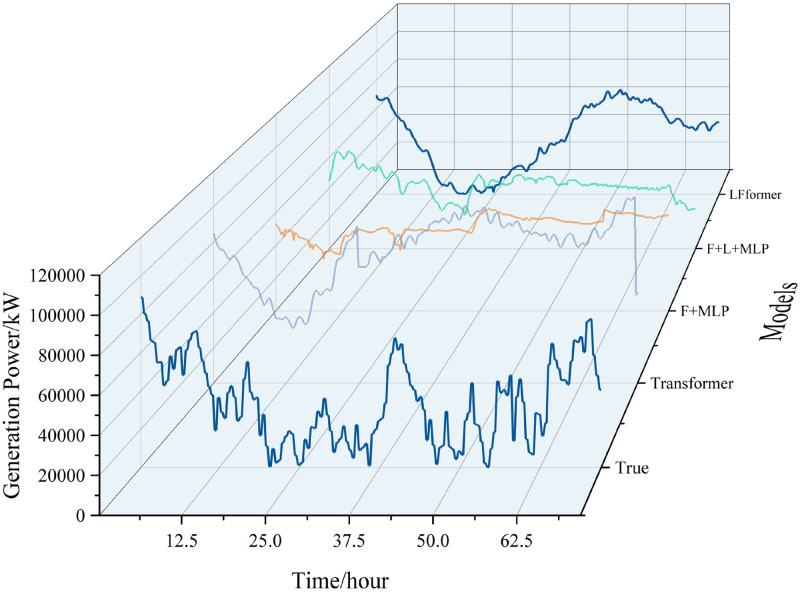
Horizontal comparison curve(a).

**Fig 8:**
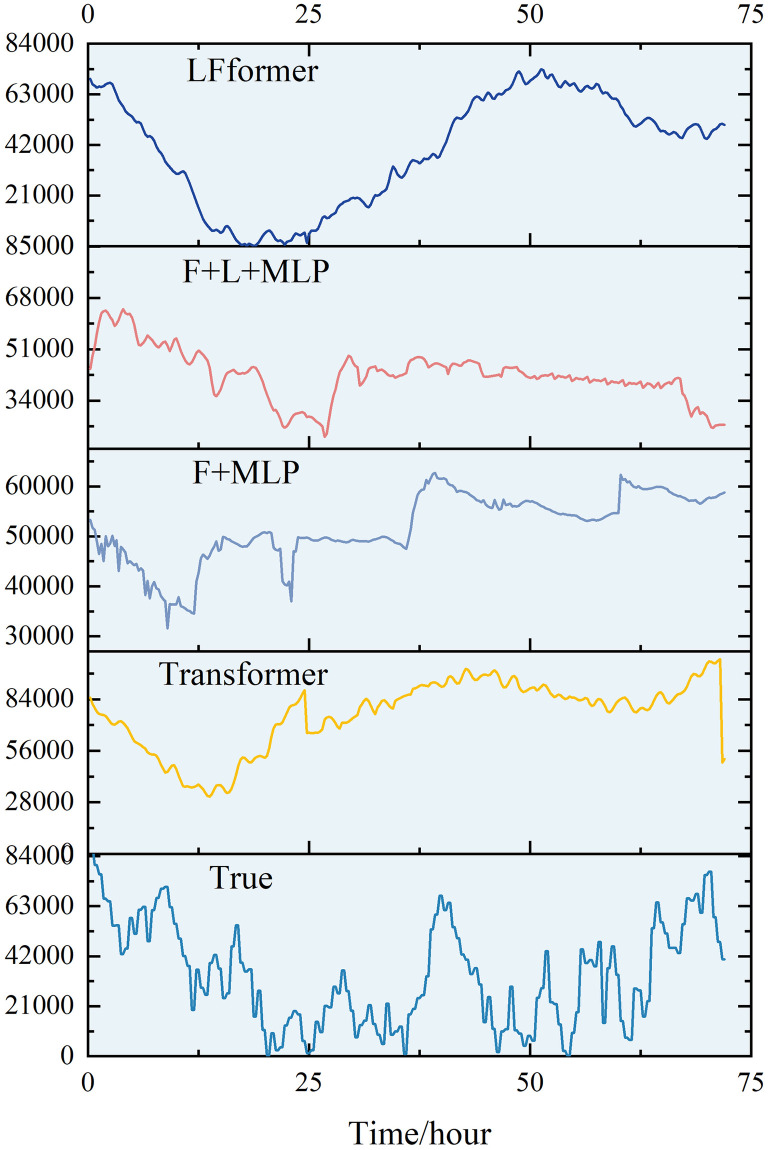
Horizontal comparison curve(b).

For the data in this paper, the horizontal comparison curves are shown in Figs 7 and 8.

### Comparative experimental results analysis

Ultra-short-term prediction with high temporal resolution can provide key information for real-time scheduling of the power system and help wind farms to schedule power generation more accurately, which is of great significance in ensuring the stable operation of the power grid as well as improving the utilization rate of wind power and investment benefits. In order to test the accuracy and stability of this paper’s model in the ultra-short-term (0–4 hours) and short-term (0–72 hours) wind power prediction tasks, the performance of this paper’s model is compared with that of other similar models on three time scales, 0–4 hours, 0–24 hours and 0–72 hours, using the evaluation metrics described in the previous section.

#### Ultra-short-term prediction

For the data in this paper, the performance of each model for ultra-short-term prediction is shown in Table 4. LFformer is the model in this paper.

**Table 4:** Performance comparison of ultra-short-term prediction.

model	P_MAPE_/%	RMSE/kW	MSE/kW	MAE/kW
Transformer	19.90	13743.47	1.89 × 10^8^	10612.43
Autoformer	35.66	23346.64	5.45 × 10^8^	19592.52
Informer	31.10	26807.42	7.19 × 10^8^	21828.03
NHits	33.55	23038.70	5.31 × 10^8^	22320.20
Performer	19.43	15955.44	2.55 × 10^8^	13265.01
Reformer	33.55	23038.70	5.31 × 10^8^	22320.20
**LFformer**	**15.97**	**11977.48**	**1.43** × **10**^**8**^	**10169.12**

Bolded data is optimal.

Analyzing the data in the table, it can be seen that through the feature extraction of Legendre polynomials and the periodicity and trend analysis of Fourier Transform, the LFformer model is able to more accurately capture the key information and patterns in the data, which improves the prediction accuracy, and thus LFformer’s *P*_*MAPE*_ is 15.97%, which is the lowest among all models, indicating that the LFformer has the best performance in prediction accuracy. In addition, the data smoothing effect of Legendre polynomials and the denoising effect of Fourier transform help to reduce the prediction error of the model, as shown in the RMSE of LFformer is 11977.48kW, the MSE is 1.43 × 10^8^kW, and the MAE is 10,169.12kW, which is the lowest among all models, which further confirms the prediction performance of LFformer. the advantages of LFformer in prediction performance.

**Fig 9:**
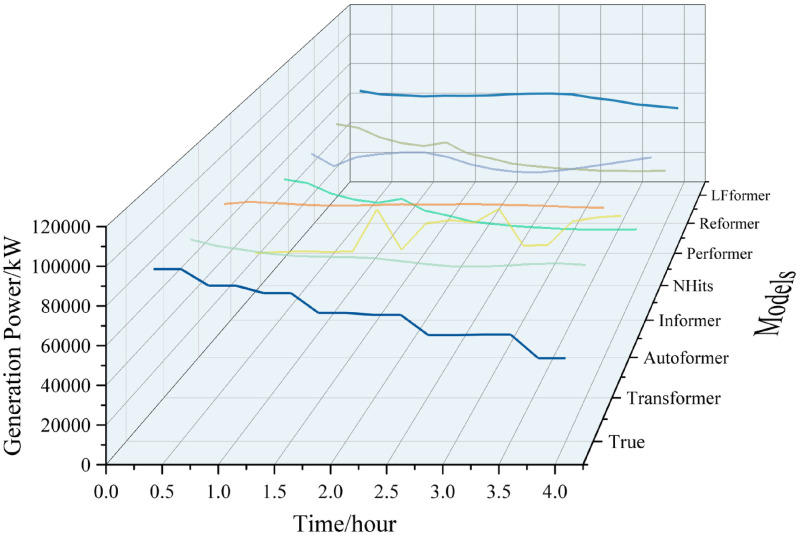
Ultra-short-term projection curves.

**Fig 10:**
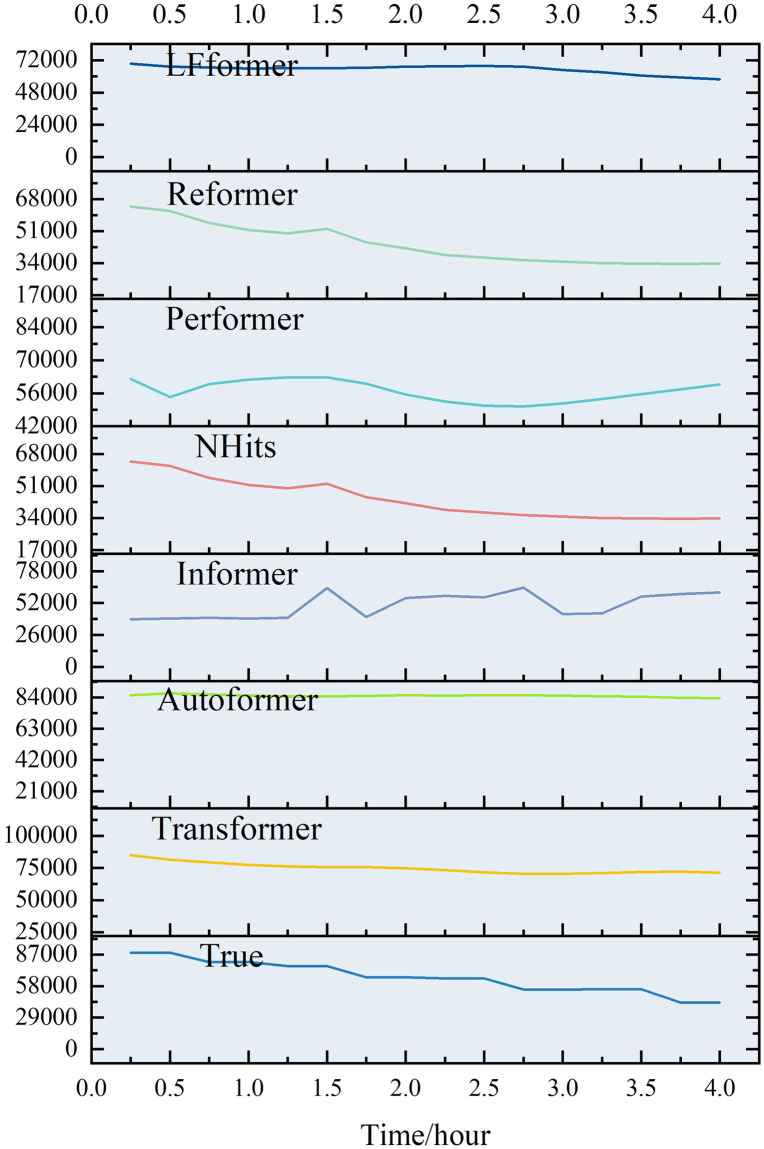
Comparison of ultra-short-term forecast curves.

For the data in this paper, the ultra-short-term prediction curves for each model are shown in Figs 9 and 10.

Analyzing the curve graph, it can be seen that the LFformer model has the highest degree of fit between the predicted and actual values, and is basically consistent with the actual curve in terms of trend, indicating that the LFformer model has an advantage in terms of prediction accuracy and trend-capturing ability. Since there is no obvious volatility or periodicity on the ultra-short-term curve, it is impossible to judge the model’s processing ability related to these aspects, and it is necessary to extend the time scale for further discussion.

#### Short-term (0–24 hours) predictions

For the data in this paper, the performance of the short-term (0–24 hours) prediction of each model is shown in Table 5. LFformer is the model in this paper.

**Table 5:** Performance comparison of Short-term (0–24 hours) prediction.

model	P_MAPE_/%	RMSE/kW	MSE/kW	MAE/kW
Transformer	480.34	29114.53	8.48 × 10^8^	20320.55
Autoformer	498.37	32788.26	1.08 × 10^9^	29569.42
Informer	103.20	28269.16	7.99 × 10^8^	23141.73
NHits	200.75	21984.90	4.83 × 10^8^	18863.89
Performer	427.63	25209.01	6.35 × 10^8^	21108.47
Reformer	200.75	21984.90	4.83 × 10^8^	18863.89
**LFformer**	**81.71**	**21511.19**	**4.63** × **10**^**8**^	**17517.71**

Bolded data is optimal.

Analyzing the data in the table, it can be seen that by fitting and transforming the data with Legendre polynomials, the LFformer model is able to capture the nonlinear features in the data more accurately, and to a certain extent, it reduces the influence of noise and outliers in the data on the prediction results, thus improving the accuracy of the prediction, so the *P*_*MAPE*_ of LFformer is 81.71%, which is the lowest among all models. In addition, the introduction of the Legendre polynomial enhances the nonlinear modeling capability of the LFformer model so that the model can better adapt to the complexity and uncertainty of the wind power data, which is demonstrated by the fact that the RMSE of the LFformer is 21511.19 kW, the MSE is 4.63 × 10^8^kW, and the MAE is 17517.71kW, which is also the lowest among all models.

**Fig 11:**
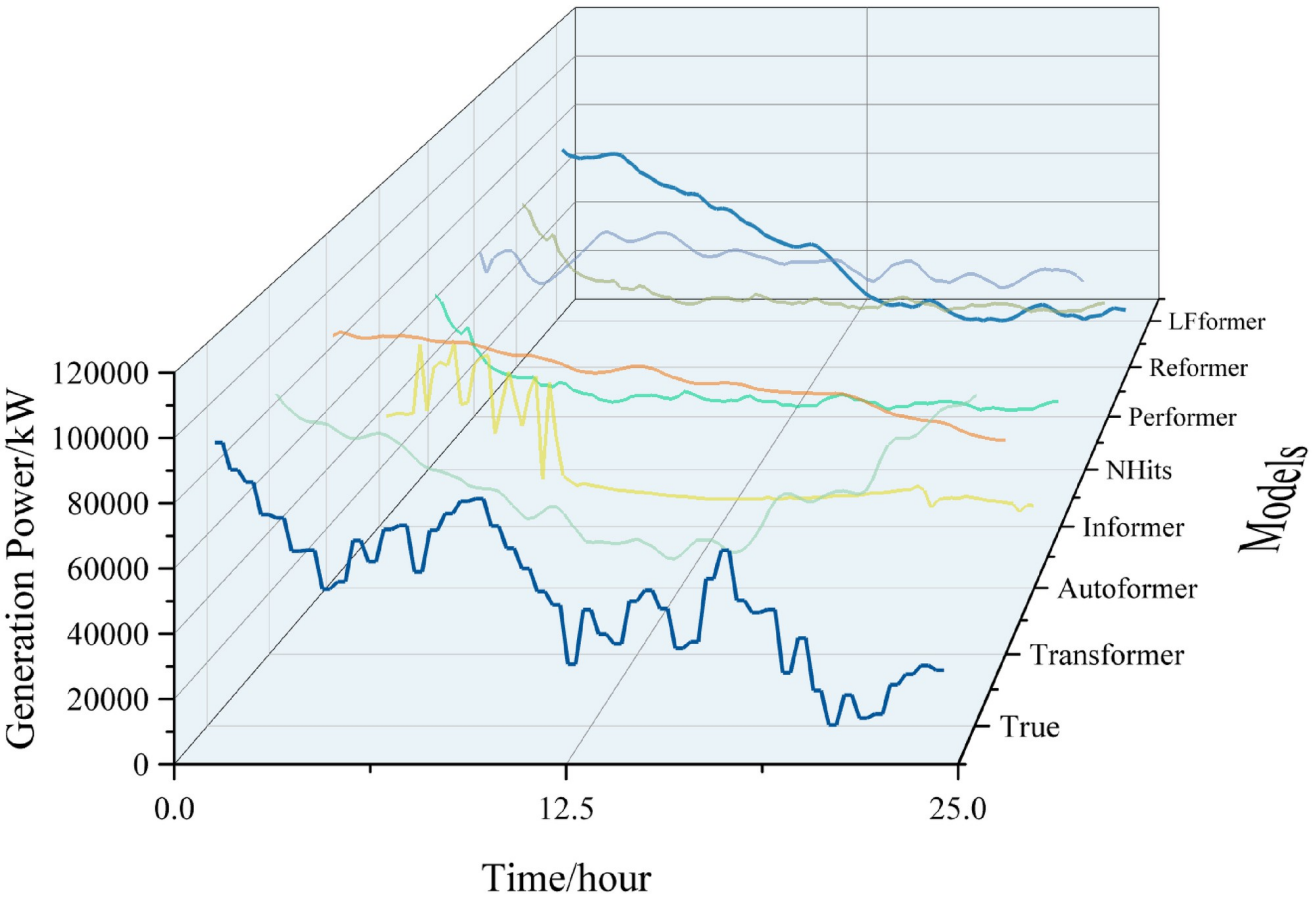
0–24 hour prediction curve.

**Fig 12:**
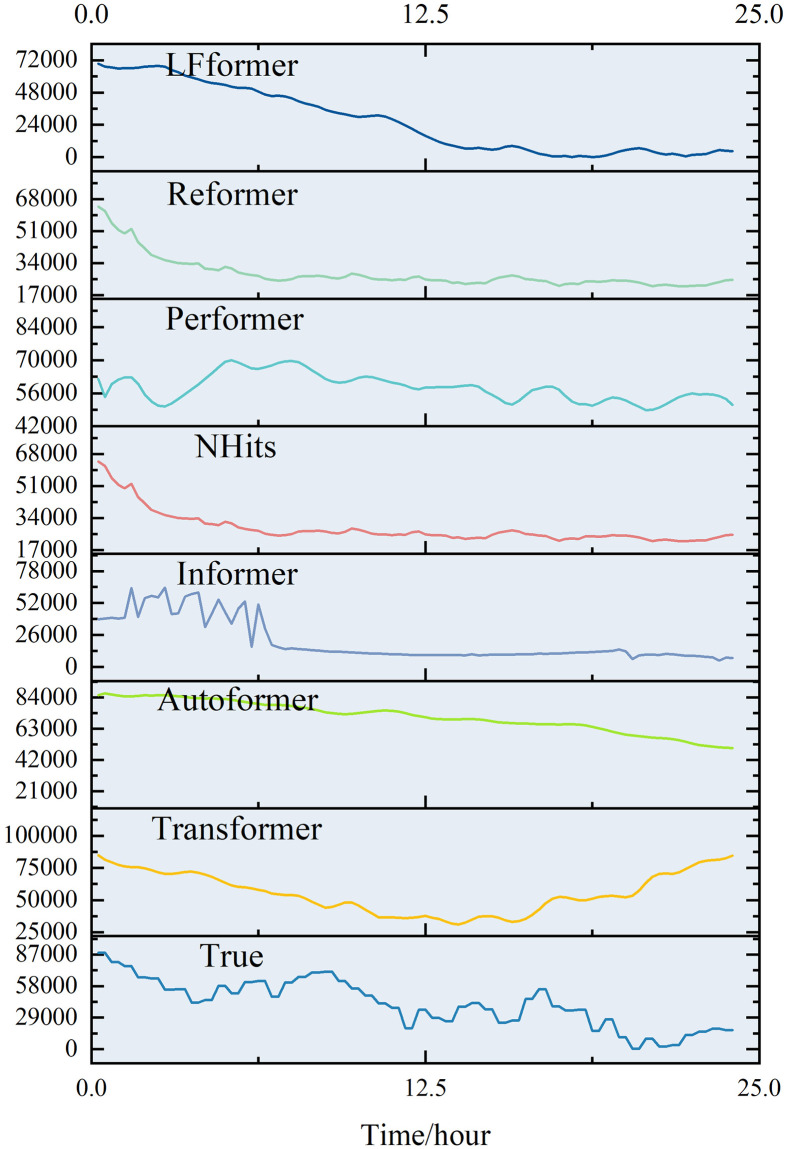
Comparison of 0–24 hour prediction curve.

For the data in this paper, the short-term (0–24 hours) prediction curves of each model are shown in Figs 11 and 12.

Analyzing the curve graph, it can be seen that the LFformer model has the highest fit between the predicted and actual values, and is basically consistent with the actual curve in terms of trend, indicating that the LFformer model has an advantage in prediction accuracy and trend capturing ability. For the actual curve volatility and periodicity, the LFformer model does not fit as well as the Transformer model at 0–15 hours. However, due to the inadequacy of the Transformer model in dealing with long series data, its prediction curve deviates from the actual curve after 15 hours.

#### Short-term (0–72 hours) predictions

For the data of this paper, the performance of short-term (0–72 hours) prediction of each model is shown in Table 6. LFformer is the model of this paper.

**Table 6:** Performance comparison of Short-term (0–72 hours) prediction.

model	P_MAPE_/%	RMSE/kW	MSE/kW	MAE/kW
Transformer	906.66	51259.11	2.63 × 10^9^	44257.91
Autoformer	427.98	26842.60	7.21 × 10^8^	23113.73
**Informer**	**305.86**	**22672.60**	**5.14** × **10**^**8**^	**17673.43**
NHits	598.98	33358.00	1.11 × 10^9^	28126.35
Performer	851.63	45661.94	2.09 × 10^9^	39502.56
Reformer	598.98	33358.00	1.11 × 10^9^	28126.35
LFformer	471.27	28092.05	7.89 × 10^8^	22024.00

Bolded data is optimal.

Although the LFformer model shows some advantages, its *P*_*MAPE*_, RMSE, MSE and MAE metrics are not optimal among all compared models. This may be due to the fact that wind power data have different characteristics on different time scales. In the 0–72 hour prediction, the data contains more uncertainties and changing factors, which increases the difficulty of prediction. In contrast, the Informer model, with its multi-layer Transformer encoder and decoder modules, is able to dig deeper into the features in the input sequences and capture the dependencies between different locations through the multi-head self-attention mechanism, which in turn better captures the long-range dependencies in the sequences, thus showing better performance on the prediction task in longer time scales.

**Fig 13:**
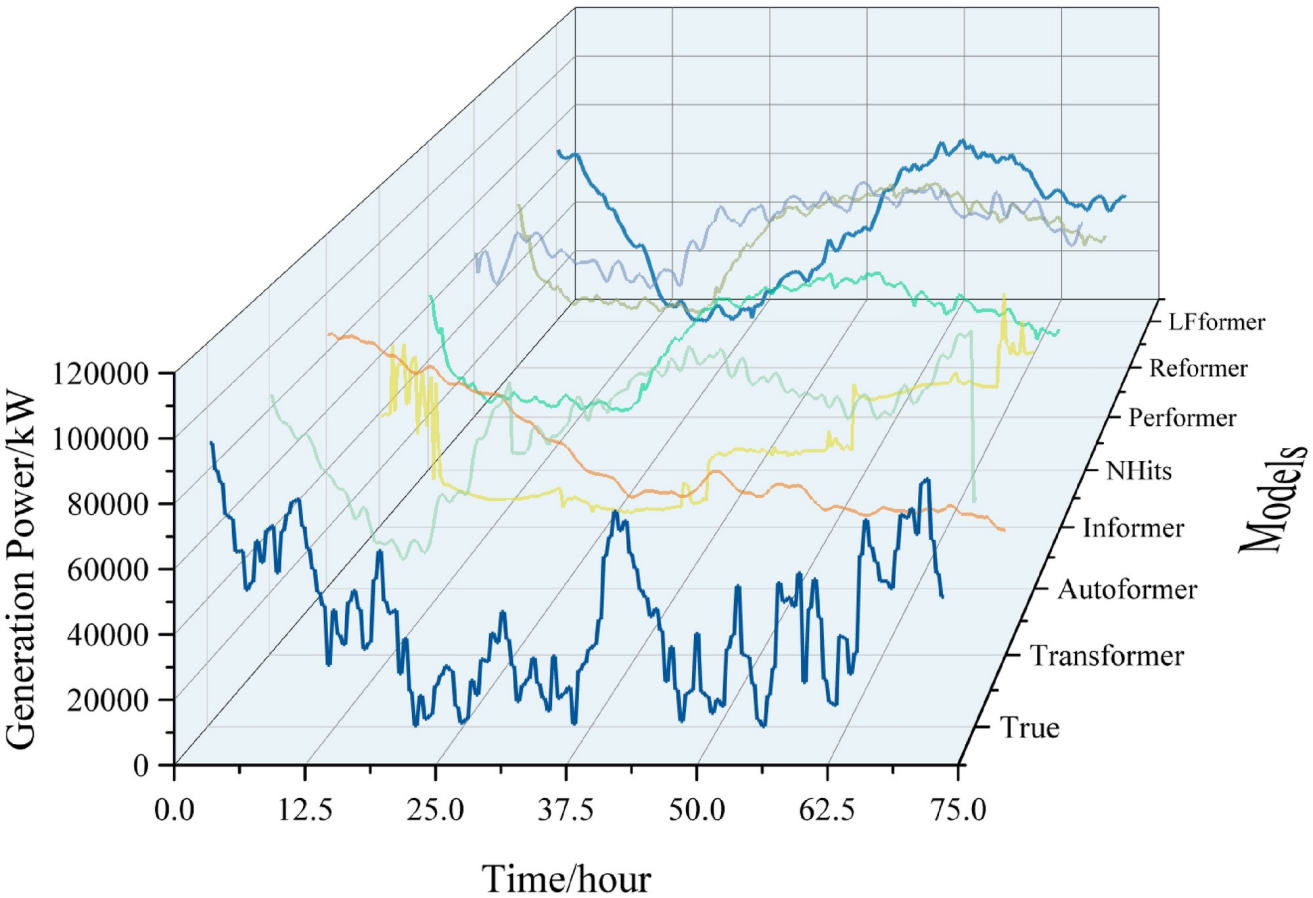
0–72 hour prediction curve.

**Fig 14:**
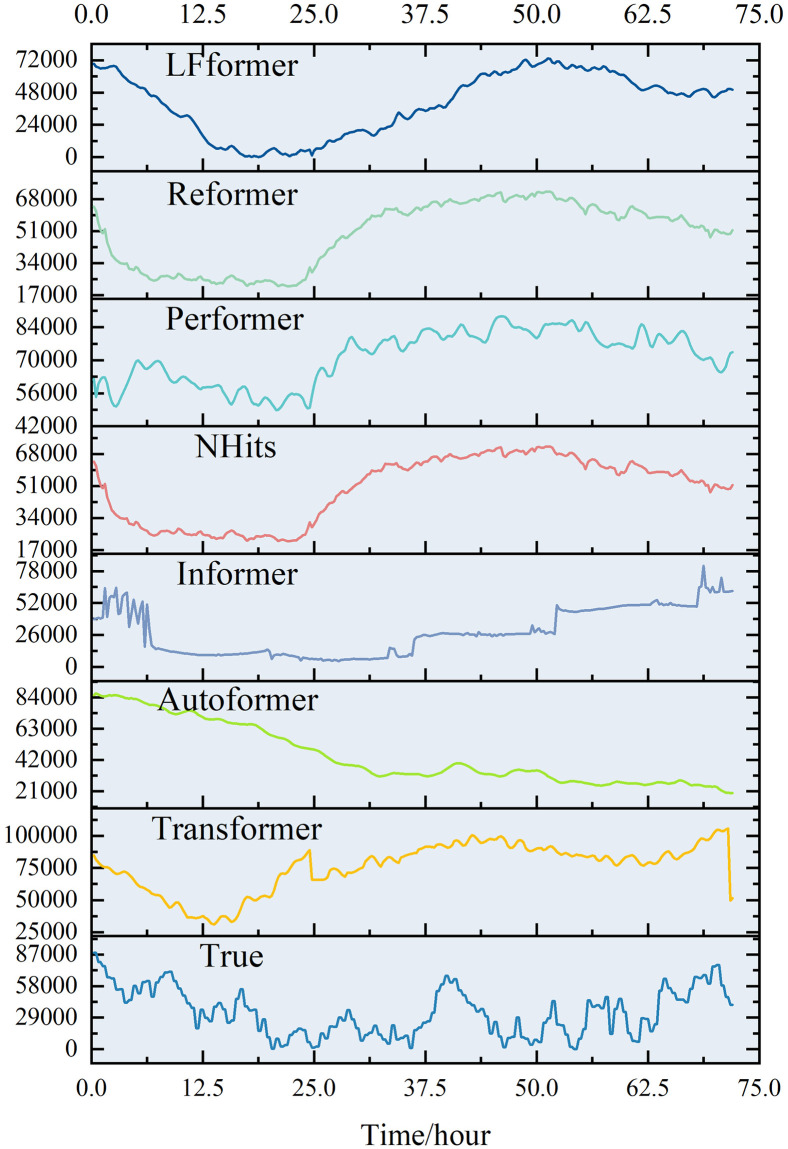
Comparison of 0–72 hour prediction curve.

For the data in this paper, the short-term (0–72 hours) prediction curves for each model are shown in Figs 13 and 14.

The predicted values of the LFformer model have a better fit with the actual values, and are basically consistent with the actual curves in terms of trend, which proves that the LFformer model has certain advantages in terms of prediction accuracy and trend capturing ability. For the volatility and periodicity of the actual curve, the LFformer model has a certain degree of fitting.

In summary, the performance of the LFformer model in the ultra-short-term and short-term (0–24 hours) prediction tasks is not optimal in the 0–72 hours prediction task due to the performance of similar models, but it is still at a high level.

## Conclusions

In this paper, a short-term prediction model of wind power generation power based on improved Transformer is proposed, and the superiority of the model in ultra-short-term wind power generation power prediction is verified through experiments and analysis. It improves the traditional prediction methods in dealing with wind power data that are complex, variable, nonlinear and contain multiple time scale features. By constructing and optimizing the model, the following research hypotheses are successfully implemented in this paper:

(1) Through the Encoder-Decoder architecture, the ability of the model to pay attention to the input sequences when generating the output can be enhanced to improve the performance and efficiency of the model in processing the sequence data.
(2) Through Legendre polynomials, with different orders of Legendre polynomials corresponding to different frequency components, the data sequences can be projected onto a bounded dimensional space for feature extraction of evolving historical data.
(3) Feature selection by Fourier transform can reduce the accumulation of historical noise and improve the performance of the model.
(4) Ultra-short-term prediction of the output of the above steps is performed by MLP, and the accuracy and stability of the prediction results are better than those of similar models.

In summary, the LFformer short-term prediction model proposed in this paper has better prediction capability than traditional methods in the short-term wind power prediction task.

## Supporting information

S1 Dataset (CSV)

S2 Dataset (CSV)
